# Protective Effect of Metformin against Hydrogen Peroxide-Induced Oxidative Damage in Human Retinal Pigment Epithelial (RPE) Cells by Enhancing Autophagy through Activation of AMPK Pathway

**DOI:** 10.1155/2020/2524174

**Published:** 2020-07-24

**Authors:** Xia Zhao, Linlin Liu, Yizhou Jiang, Marta Silva, Xuechu Zhen, Wenhua Zheng

**Affiliations:** ^1^Center of Reproduction, Development & Aging and Institute of Translation Medicine, Faculty of Health Sciences, University of Macau, Taipa, Macau, China; ^2^Jiangsu Key Laboratory of Neuropsychiatric Diseases and College of Pharmaceutical Sciences, Soochow University, Suzhou, Jiangsu 215123, China

## Abstract

Age-related macular degeneration (AMD) is a leading cause of blindness with limited effective treatment. Although the pathogenesis of this disease is complex and not fully understood, the oxidative damage caused by excessive reactive oxygen species (ROS) in retinal pigment epithelium (RPE) has been considered as a major cause. Autophagy is essential for the degradation of cellular components damaged by ROS, and its dysregulation has been implicated in AMD pathogenesis. Therefore, strategies aiming to boost autophagy could be effective in protecting RPE cells from oxidative damage. Metformin is the first-line anti-type 2 diabetes drug and has been reported to stimulate autophagy in many tissues. We therefore hypothesized that metformin may be able to protect RPE cells against H_2_O_2_-induced oxidative damage by autophagy activation. In the present study, we found that metformin attenuated H_2_O_2_-induced cell viability loss, apoptosis, elevated ROS levels, and the collapse of the mitochondria membrane potential in D407 cells. Autophagy was stimulated by metformin, and inhibition of autophagy by 3-methyladenine (3-MA) and chloroquine (CQ) or knockdown of Beclin1 and LC3B blocked the protective effects of metformin. In addition, we showed that metformin could activate the AMPK pathway, whereas both pharmacological and genetic inhibitions of AMPK blocked the autophagy-stimulating and protective effects of metformin. Metformin conferred a similar protection against H_2_O_2_-induced oxidative damage in primary cultured human RPE cells. Taken together, these results demonstrate that metformin could protect RPE cells from H_2_O_2_-induced oxidative damage by stimulating autophagy via the activation of the AMPK pathway, supporting its potential use in the prevention and treatment of AMD.

## 1. Introduction

Age-related macular degeneration (AMD) is the leading cause of blindness in individuals over 50 years of age. It is a disease that affects the macula of the retina, causing a chronic and progressive vision loss [1]. Late AMD can be divided into neovascular (wet) and nonneovascular (dry) forms [1]. Currently, therapies such as antivascular endothelial growth factor (anti-VEGF) therapy have been proven to be effective in treating wet AMD [2]. However, dry AMD, which accounts for approximately 90% of AMD cases, still lacks an effective treatment.

Although the pathogenesis of dry AMD is complex, the degeneration of the aging retinal pigment epithelium (RPE) cells is widely considered to be the initial event [3]. The RPE consists of a single layer of epithelial cells that sustains the function of photoreceptor cells by supporting the phagocytosis of photoreceptor outer segments (POS), vitamin A metabolism, and the regeneration of visual pigments [4–7]. Its impairment results in a secondary degradation of photoreceptors and eventually leads to vision loss [3, 8]. RPE cells are especially susceptible to ROS-induced oxidative damage. As a high energy-demanding tissue, RPE cells produce high levels of ROS derived from the oxygen metabolism [5, 9]. ROS can also be generated by light or the phagocytosis of POS in RPE [5, 10]. Additionally, studies have been showing that RPE cell impairment can lead to the accumulation of damaged organelles and various nonfunctional or toxic proteins, including lipofuscin, and promote the formation of drusen which is a typical characteristic of AMD [11].

Autophagy is a protective mechanism designed for the degradation and removal of different cellular components, including those damaged by ROS, supporting cellular renovation and homeostasis [5]. The autophagic process begins with the formation of a sequestering membrane, termed as phagophore, to form an autophagosome that subsequently engulfs the intracellular components and carry them to lysosomes for degradation [12]. Autophagy initiation is controlled by the activation of ULK-1 complex I (contains ULK1, FIP200, and ATG13) and of Beclin1 complex II (contains the proteins p150 and Atg14L and class III phosphatidylinositol 3-phosphate kinase Vps34). Following amino acid withdrawal, ULK-1 was shown to phosphorylate Beclin1, and this phosphorylation step is crucial for the function of Beclin1 in autophagy [13]. The active ULK-1 and Beclin1 complexes relocalize to the site of autophagosome initiation, the phagophore, where they both contribute to the recruitment of different downstream autophagy components [14]. The phagophore formation is followed by the elongation stage of autophagy essential for the expansion of the autophagosomal membrane which requires ATG5–ATG12 and the microtubule-associated protein light chain 3 (LC3–ATG8) conjugation systems [15]. The autophagosomal formation is denoted by the combination of cytosolic LC3-I with phosphatidylethanolamine on the surface of the autophagosomal membrane forming LC3-II protein, which is visible as discrete puncta on immunofluorescence analysis. Being proportional to the number of autophagic vesicles, LC3 is considered an autophagosome marker molecule [16]. The LC3-binding protein p62, also known as sequestosome 1 (SQSTM1), binds to the LC3-II protein located on the inner membrane of the autophagosome and then is degraded in autophagolysosomes. The inhibition of autophagy is accompanied by the accumulation of p62 protein [17]. Many lines of evidences suggest that autophagy impairment is associated with a variety of diseases, such as diabetes and cancer, infectious diseases, and neurodegenerative diseases, including AMD [18, 19]. Specifically, autophagy has been shown to be crucial for RPE homeostasis and visual cycle, as evidenced by the decreased POS responses to light stimuli in mice with essential autophagy gene Atg5-deficient RPE cells [20]. In AMD, impaired autophagic capacity occurs simultaneously with the accumulation of dysfunctional mitochondria and toxic proteins that ultimately lead to RPE cell damage or apoptosis [7, 21]. While autophagy inhibition in ARPE-19 cells by 3-MA or by the knockdown of ATG7 or Beclin1 was reported to increase its susceptibility to H_2_O_2_-induced oxidative damage, autophagy upregulation by rapamycin was shown to protect the cells from H_2_O_2_-induced injury [7]. Therefore, strategies aimed at upregulating autophagy may be helpful for protecting RPE cells from oxidative damage and preventing or reversing the progression of AMD.

Metformin, a biguanide derivate widely used in the treatment of type 2 diabetes, has been shown to stimulate autophagy in a variety of tissues by activating AMP-activated protein kinase (AMPK) [22–24]. AMPK is an energy sensor that maintains the cellular energy homeostasis by regulating several important cellular processes, such as cell growth and proliferation, apoptosis, and autophagy [22, 25]. AMPK can activate autophagy by inhibiting the mammalian target of rapamycin (mTOR) [26]. In addition, AMPK activation also promotes autophagy by directly phosphorylating ULK1, the protein kinase that initiates autophagy [27]. We have previously reported that metformin could protect neuronal cells from H_2_O_2_-induced oxidative damage by activating AMPK [28]. Given the crucial role of autophagy in AMD pathogenesis, we therefore hypothesized that metformin may confer a similar protection in RPE cells by stimulating autophagy. In the present study, we aimed to investigate whether metformin could protect RPE cells from H_2_O_2_-induced oxidative damage and whether the protective effects are associated with the upregulation of autophagy promoted by AMPK activation.

## 2. Materials and Methods

### 2.1. Materials

Human retinal pigment epithelial cell line D407 was obtained from the cell bank, Sun Yat-sen University (Guangzhou, China); human primary cultured human retinal pigment epithelial (hRPE) cells were obtained from the State Key Laboratory of Ophthalmology, Zhongshan Ophthalmic Center, and were approved by the Ethics Committee of Zhongshan Ophthalmic Center (2018KYPJ082, 15 May 2018). Analytical grade metformin was purchased from Meilunbio (Dalian China). H_2_O_2_ and DMSO were obtained from Sigma (St. Louis, MO, USA). LDH Cytotoxicity Assay Kit was ordered from Beyotime Biotechnology (Haimen, China). Fetal bovine serum (FBS) and DMEM were purchased from Invitrogen (Carlsbad, CA, USA); methyl thiazolyl tetrazolium (MTT), JC-1, and Hoechst 33342 were purchased from Molecular Probes (Eugene, OR, USA). DCFH-DA reagent was ordered from Beyotime Institute of Biotechnology (Shanghai, China). Annexin V-FITC/PI Apoptosis Detection Kit was obtained from BD Biosciences (San Diego, CA, USA). Dorsomorphin (compound C), 3-methyladenine (3-MA), and chloroquine (CQ) were ordered from Calbiochem (San Diego, CA, USA). Antibodies were purchased from Cell Signaling Technology (Woburn, USA). The detailed information of the antibodies is shown in Table 1. Tandem fluorescent-tagged LC3 (mRFP-GFP-LC3) reporter, shRNA for LC3B, Beclin1, and AMPK shRNA were synthesized by Shanghai Genechem Co. Ltd (Shanghai, China). The shRNA sequence information is shown in Table 2.

### 2.2. Cell Culture and Treatments

D407 cells were maintained in DMEM supplemented with 10% FBS and 1% penicillin/streptomycin. Human RPE cells (hRPE) were cultured in DMEM/F12 supplemented with 10% FBS and 1% penicillin/streptomycin. Cell cultures were incubated in a 37°C incubator with 5% CO_2_ humidified atmosphere. The medium was replaced every 2-3 days and subcultured using 0.25% trypsin treatment twice a week. Passages 6-8 of D407 cells were used for all the experiments. Cell treatments were performed by incubating the cells with the respective drugs diluted in blank cell culture medium. In cells treated with more than one drug, the medium was changed and substituted by the subsequent medium-containing compound.

### 2.3. MTT Assay

Cell viability was estimated using MTT assay. Briefly, cells were seeded into 96-well plates at a density of 1 × 10^4^ cells/mL. On the following day, cells were incubated with drugs diluted in cell culture medium for an appropriate time. Cells were then incubated with MTT (0.5 mg/mL) for additional 3 h, and the medium was replaced with DMSO (100 *μ*L/well) to dissolve the formazan crystals. The absorbance at 570 nm was measured using Infinite® 200 PRO NanoQuant Multimode Microplate Reader (Tecan Trading AG, Switzerland). Assays were repeated at least three times in quintuplicate.

### 2.4. LDH Assay

Cell cytotoxicity was assessed by measuring the activity of lactate dehydrogenase (LDH) released into the medium. Upon damage of the cell membranes, LDH is released into the culture medium and its quantification can be used as a measure of cell death. Briefly, D407 cells were seeded into 96-well plates at a density of 1 × 10^4^ cells/mL, and the activity of LDH was determined using LDH assay (C0017, Beyotime, Shanghai, China) according to the manufacturer's instructions. The absorbance value was measured by the Infinite® 200 PRO NanoQuant Multimode Microplate Reader at a wavelength of 490 nm.

### 2.5. Measurement of Intracellular ROS Levels

Intracellular ROS levels were evaluated using the DCFH-DA reagent (S0033, Beyotime, Shanghai, China). After appropriate treatment, D407 cells were washed with 1x PBS for 3 times and then incubated with 5 mM DCFH-DA reagent in blank DMEM medium for 1 h in the dark and then rinsed twice with 1xPBS. The absorbance value was measured by the Infinite® 200 PRO NanoQuant Multimode Microplate Reader at an excitation wavelength of 488 nm and an emission wavelength of 525 nm. The image was taken using a fluorescent microscope (Olympus, Japan). ROS levels were quantified using ImageJ software. All values of % ROS levels were normalized to the control group.

### 2.6. Measurement of Mitochondrial Membrane Potential (*Δψ*m)

JC-1 dye was used to measure the mitochondrial membrane potential (*Δψ*m). The cells were seeded into 96-well plates (1 × 10^4^ cells/well). After appropriate treatment, cells were incubated with JC-1 dye (10 *μ*g/mL in medium) at 37°C for 30 min and then washed twice with PBS. The red (excitation 560 nm, emission 595 nm) and green (excitation 485 nm, emission 535 nm) fluorescence signals were monitored using the Infinite® 200 PRO NanoQuant Multimode Microplate Reader. The ratio of JC-1 red/green fluorescence intensity was calculated.

### 2.7. TUNEL Staining

Apoptosis was determined by the TUNEL assay using the TUNEL kit (C1089, Beyotime, Shanghai, China). The cells were seeded into 96-well plates (1 × 10^4^ cells/well). After treatment, cells were incubated with a TUNEL reaction mixture (50 *μ*L) at 37°C for 60 min in the dark condition. Cells staining red were positive for TUNEL staining and considered apoptotic. The apoptotic index was measured according to the following formula: apoptotic index (%) = (apoptotic cells/total cells) × 100%.

### 2.8. Hoechst 33342 Staining

The DNA binding dye Hoechst 33342 was used to measure cell apoptosis. The cells were seeded into 96-well plates (1 × 10^4^ cells/well). After treatment, cells were washed with PBS and fixed with 4% formaldehyde for 15-20 min at room temperature. The cells were then incubated with 10 *μ*g/mL of Hoechst 33342 for 2 min at room temperature. After this period, the cells were washed with PBS and observed under EVOS FL Imaging System (Thermo Fisher Scientific, Waltham, MA, USA). Cells exhibiting fragmented nuclei or condensed chromatin were scored as apoptotic cells.

### 2.9. Flow Cytometry

Flow cytometry was used to assess cell apoptosis. Briefly, D407 cells were trypsinized after the treatment, washed twice with 1x PBS, and then centrifuged at 1000 rpm for 5 min and resuspended in Annexin V-FITC 1x binding buffer (195 *μ*L). The cells were supplemented with Annexin V-FITC (5 *μ*L) and incubated in the dark at 37°C for 20 min. After this period the cells were centrifuged at 1000 rpm for 5 min, resuspended in 1x binding buffer (190 *μ*L) and incubated with propidium iodide (PI) (10 *μ*L) in the dark for 5 min. Apoptosis was quantified using the flow cytometer. Cell Quest Pro software (BD AccuriC6, BD, USA) was used for the analysis of the apoptosis condition.

### 2.10. Immunocytochemistry

After appropriate treatment, D407 cells were fixed with 4% paraformaldehyde (PFA) at room temperature for 15 min and then washed with 1x PBS for three times. The cells were then incubated with 1x PBST (containing 0.25% Triton X-100) for 10 min and blocked with 3% BSA for 1 h at room temperature. Afterwards, cells were incubated with the primary antibody in PBS containing 1% BSA at 4°C overnight. The following day, the cells were washed 3 times with PBS for 5 min and incubated with the secondary antibody for 1 h at room temperature in the dark. Nuclei were counterstained with one drop of antifade mounting medium with DAPI (P0131, Beyotime), and the images were acquired with a Nikon A1 confocal microscope.

### 2.11. Measurement of the Autophagic Flux

Monitoring Autophagy flux is important for the analysis of autophagy. A tandem fluorescent-tagged LC3 (mRFP-GFP-LC3) virus vector was used to investigate the autophagic flux based on the different pH stability of mRFP and GFP fluorescent proteins. While mRFP is pH insensitive, GFP is degraded in the acidic environment of autophagolysosomes. Cells with no autophagy and cells containing autophagosomes exhibit yellow fluorescence due to the coexpression of mRFP and GFP. Upon fusion of autophagosomes and lysosomes to form autophagolysosomes, the acidic lysosomal environment quenches the acid-sensitive GFP, while RFP is not affected, causing autophagolysosomes to exhibit a red fluorescence. Therefore, red fluorescence can be used to measure the autophagic flux. Approximately 5 × 10^4^ D407 cells were seeded in 12-well plates covered with coverslips and grown in DMEM medium with 10% FBS until 60% confluence. Diluted virus stock solutions in blank medium (20, 40, and 80 multiplicity of cellular infection (MOI)) were added to the cells. After 12 h of infection, the medium was changed to complete culture medium (DMEM medium with 10% FBS) and the cells were cultured for additional 48 h before adding the drugs. After treatment, the cells were fixed in 4% PFA for 10-15 min at room temperature. Fixed cells were then washed twice with 1x PBS. The coverslips were mounted on glass slides using VECTASHIELD® Mounting Medium without DAPI (H-1000). The images were acquired using a Nikon A1 confocal microscope.

### 2.12. Western Blotting

Cells were harvested and lysed with ice-cold RIPA lysis buffer. Protein concentration was determined by the BCA method. Samples with an equal amount of proteins (50 *μ*g) were separated by 10% SDS-PAGE and then transferred to a PVDF membrane. After blocking with 3% BSA for 1 h, the membranes were incubated with the selective primary antibodies at 4°C overnight. The next day, the primary antibody was washed with 1x TBST for three times and incubated with a secondary antibody for another 2 h. The intensity of the bands was quantified using ImageJ software.

### 2.13. LC3B And AMPK Silencing by shRNA

Gene silencing by shRNA was performed as previously described [29]. Before the experiment, transfection complexes were made by mixing shRNA with Lipo2000 and Opti-MEM followed by incubation for 15 min at room temperature. Obtained transfection complexes were added drop-wise onto cell cultures. The cells were then moved to a 37°C incubator and maintained for 6 h. After this period, the medium was replaced by complete medium. Cells were collected for further experiments 48 h after transfection.

### 2.14. Statistical Analysis

All the results are presented as the mean ± SD for triplicates. Data was analyzed using GraphPad Prism 5.0 statistical software (GraphPad software, Inc., San Diego, CA, USA). Statistical analysis was carried out using one-way ANOVA followed by Tukey's multiple comparison post hoc test, with *p* < 0.05 being considered statistically significant.

## 3. Results

### 3.1. Metformin Protected D407 Cells from H_2_O_2_-Induced Cell Death

**Figure 1:**
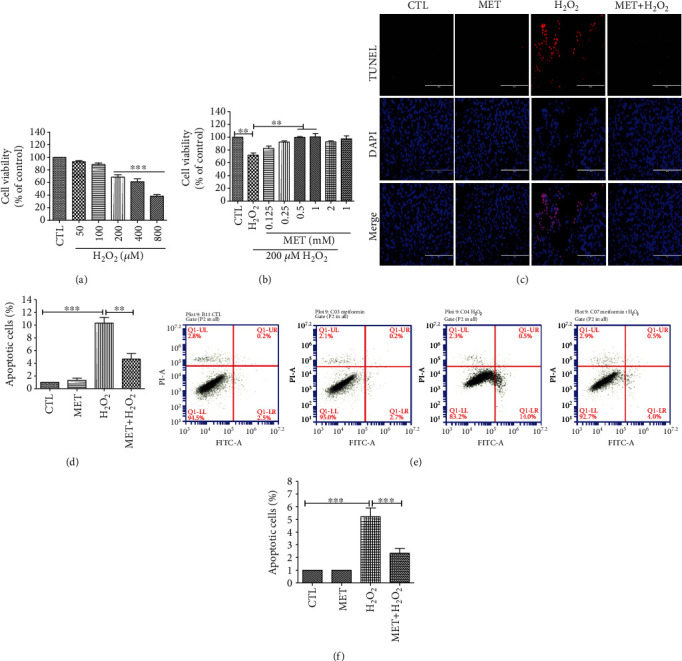
Metformin attenuated the decrease in cell viability and apoptosis induced by H_2_O_2_ in D407 cells. (a) Cells were treated with H_2_O_2_ (50-800 *μ*M) for 24 h, and cell viability was measured using the MTT assay. (b) Cells were pretreated with metformin (MET) at the indicated concentrations and then incubated with or without 200 *μ*M H_2_O_2_ for further 24 h. Cell viability was measured using the MTT assay. (c) Cells were pretreated with 1 mM metformin and then incubated with or without 200 *μ*M H_2_O_2_ for further 24 h. Micrographs of representative cultures measured by TUNEL staining (scale bars, 200 *μ*m). (d) Quantitative analysis of (c). (e) Graphs of representative cultures measured by flow cytometry. (f) Quantitative analysis of (e). Data is presented as the mean ± SD; ^∗∗^*p* < 0.01, ^∗∗∗^*p* < 0.001.

To evaluate the cytotoxicity of H_2_O_2_, D407 cells were incubated with varying concentrations of H_2_O_2_ (50-800 *μ*M) for 24 h. As shown in Figure 1(a), H_2_O_2_ significantly decreased the cell viability in a concentration-dependent manner starting at 200 *μ*M, and this concentration was chosen for the following experiments. To evaluate the protective effect of metformin on H_2_O_2_-induced cell toxicity, D407 cells were pretreated with metformin (0.125-1 mM) for 2 h before being exposed to 200 *μ*M H_2_O_2_ for 24 h. The results of the MTT assay showed that metformin pretreatment significantly attenuated H_2_O_2_-induced cell viability loss (Figure 1(b)). As 1 mM of metformin showed no cytotoxicity and also the most significant protection, this concentration was used in subsequent experiments. Cell apoptosis plays an essential role in H_2_O_2_-induced cell injury and metformin pretreatment significantly mitigated cell apoptosis caused by H_2_O_2_. Metformin treatment alone did not induce any detectable apoptosis in D407 cells (Figures 1(c)–1(f)).

### 3.2. Metformin Inhibited H_2_O_2_-Induced Reactive Oxygen Species (ROS) Production and Loss of Mitochondrial Membrane Potential (*Δψ*m) in D407 Cells

**Figure 2:**
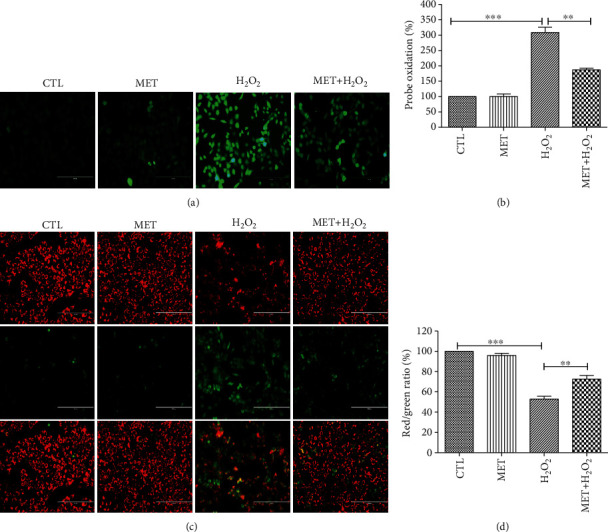
Metformin inhibited H_2_O_2_-induced increase of ROS levels and restored the mitochondria membrane potential in D407 cells. Cells were pretreated with 1 mM metformin and then incubated with or without 200 *μ*M H_2_O_2_ for further 24 h. (a) Micrographs of fluorescent labeled cells stained by DCFH-DA reagent for ROS (scale bars, 200 *μ*m). (b) Quantitative analysis of (a). (c) The decline of the membrane potential was reflected by the shift of fluorescence from red to green indicated by JC-1 (scale bars, 200 *μ*m). (d) Quantitative analysis of (d). Data are presented as the mean ± SD; ^∗∗^*p* < 0.01, ^∗∗∗^*p* < 0.001.

The generation of excessive ROS has been considered a major cause of H_2_O_2_-induced oxidative damage and cell death. As shown in Figures 2(a) and 2(b), 200 *μ*M H_2_O_2_ resulted in a remarkable increase of cellular ROS levels, whereas metformin treatment alone did not cause any detectable change. However, pretreatment of D407 cells with 1 mM metformin significantly inhibited H_2_O_2_-induced excessive ROS accumulation. The loss of mitochondrial membrane potential (*Δψ*m) due to mitochondrial damage is reported to be a hallmark of cell apoptosis caused by H_2_O_2_, and pretreatment of the cells with 1 mM metformin prevented the loss of *Δψ*m induced by 200 *μ*M H_2_O_2_. Metformin treatment alone did not affect the *Δψ*m in D407 cells (Figures 2(c) and 2(d)).

### 3.3. Metformin Activates Autophagy in D407 Cells

**Figure 3:**
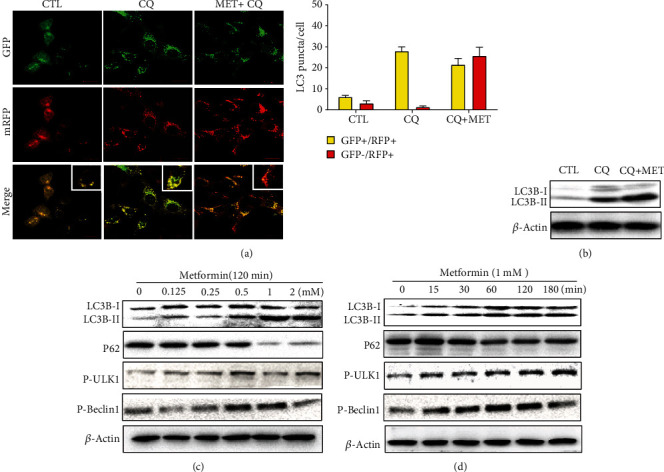
Metformin activated the autophagy signaling pathway in D407 cells. (a) Cells were transfected with tandem fluorescent-tagged LC3 reporter and treated with 60 *μ*M CQ and 1 mM metformin plus 60 *μ*M CQ for 2 h. Images were taken by a confocal microscope (scale bars, 20 *μ*m). (b) D407 cells were treated with 60 *μ*M CQ and 1 mM metformin plus 60 *μ*M CQ for 2 h. The expression of LC3B-I and LC3B-II was assessed by Western blot. (c) D407 cells were treated with different concentrations of metformin for 2 h, and the expression of LC3B, p62, P-Beclin1, and P-ULK1 was assessed by Western blot. (d) D407 cells were treated with 1 mM metformin for the periods of time indicated. The expression of LC3B, p62, P-Beclin1, and P-ULK1 was assessed by Western blot.

Knowing that autophagy is an important protective mechanism to remove damaged cellular components and protect the cells from oxidative damage, we postulated that metformin could confer protection against H_2_O_2_-induced oxidative damage in D407 cells by enhancing autophagy. To test this hypothesis, we first evaluated if metformin could stimulate autophagy by measuring the autophagic flux. We first explored the optimal MOI value of the virus solution by comparing the fluorescence intensity of cells infected with virus solutions with different MOI values (20, 40, and 80) (Fig. S1). According to the results, 80 MOI had the highest virus infection efficiency and this dilution was used to treat the cells before the treatments. The autophagic flux was measured by comparing the abundance of autophagosomes labeled with yellow and red fluorescence. As shown in Figure 3(a), 1 mM metformin induced an increase in the number of red light spots indicative of the stimulatory effect of metformin on the autophagic flux in D407 cells. When autophagy occurs, the cytoplasmic form LC3-I combines with phosphatidylethanolamine to form LC3-II, which is a marker of autophagosome formation. As an isoform of LC3, LC3B is widely used as a molecular marker of autophagy. Accordingly, Western blot analysis confirmed that metformin treatment increased the level of LC3B-II expression (Figure 3(b)). Further confirmation was achieved through the assessment of the effect of metformin treatment in LC3B-II and p62 proteins levels and in ULK-1 and Beclin1 phosphorylation. As shown in Figures 3(c) and 3(d), metformin treatment promoted the increase of LC3B-II levels and of ULK-1 and Beclin1 phosphorylation in a time- and dose-dependent manner. In contrast, p62 levels were decreased by metformin treatment in a similar manner. Taken together, these findings are indicative that metformin is able to activate autophagy.

### 3.4. Autophagy Inhibition Blocked the Protective Effect of Metformin

**Figure 4:**
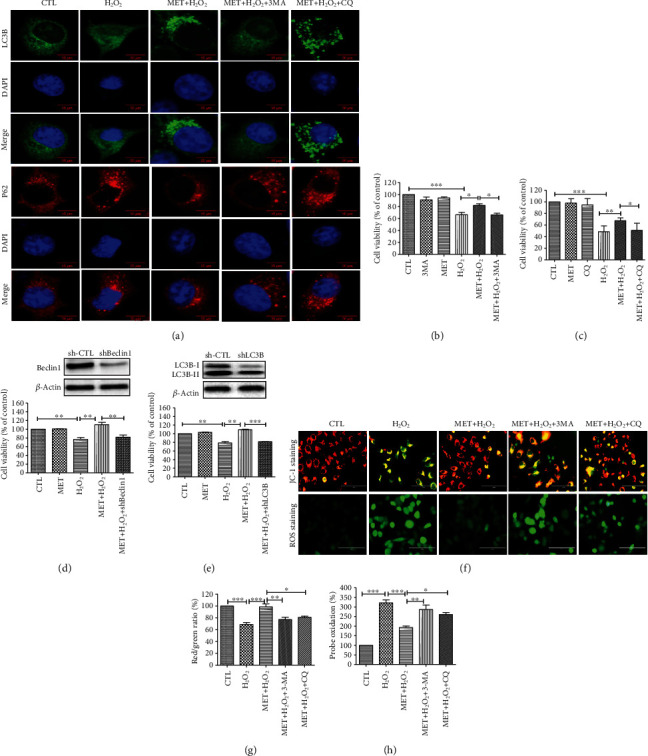
The protective effect of metformin was inhibited by autophagy blockage. D407 cells were pretreated with or without 2 mM 3-MA or 50 *μ*M CQ for 1 h before 1 mM metformin treatment for 2 h and then incubated with or without 200 *μ*M H_2_O_2_ for further 24 h. (a) The effect of 3-MA or CQ pretreatment on the expression of LC3B and p62 was assessed using ICC (scale bars, 5 *μ*m). (b) Assessment of the effect of 3-MA pretreatment on cell viability was assessed using the MTT assay. (c) Assessment of the effect of CQ pretreatment on cell viability was assessed using the MTT assay (d) D407 cells were incubated with shBeclin1 plasmid for 24 h before 1 mM metformin treatment for 2 h and then incubated with or without 200 *μ*M H_2_O_2_ for further 24 h. Cell viability was assessed using the MTT assay. (e) D407 cells were incubated with shLC3B plasmid for 24 h before 1 mM metformin treatment for 2 h and then incubated with or without 200 *μ*M H_2_O_2_ for further 24 h. Cell viability was assessed using the MTT assay. (f) The effect of Beclin1 or LC3B knockdown on the oxidative damage was assessed by JC-1 and ROS staining (scale bars, 100 *μ*m). (g, h) Quantitative analysis of (d). Data are presented as the mean ± SD; ^∗^*p* < 0.05, ^∗∗^*p* < 0.01, and ^∗∗∗^*p* < 0.001.

In order to investigate whether autophagy is involved in the metformin-mediated protection against H_2_O_2_-induced oxidative damage, we used 3-MA and CQ to inhibit the formation of functional autophagosomes and the autophagosome-lysosome fusion, respectively [30, 31]. As shown in Figure 4(a), metformin pretreatment induced an increase in the number of autophagosome puncta containing LC3B in the cells exposed to H_2_O_2_ damage, which is indicative of its ability to stimulate the autophagic flux. Acute H_2_O_2_ treatment was previously reported to induce autophagy as a protective attempt to prevent oxidative damage in RPE cells [7]. Consistent with this finding, our results showed that treating D407 cells with 200 *μ*M H_2_O_2_ for 24 h resulted in an increase of the number of LC3 puncta per cell that was further strengthened by 1 mM metformin pretreatment. p62 is a substrate of autophagy widely used as a marker of autophagic degradation [32]. A recent study has shown that oxidative stress upregulated p62 gene expression to assist selective autophagy, serving as an early protective response [33]. In line with this, we found that H_2_O_2_ induced the elevation of p62 levels in D407 cells. In addition, p62 levels were significantly decreased upon metformin treatment. 3-MA pretreatment reversed metformin-induced increase of LC3B expression and decrease of p62 expression, whereas CQ pretreatment resulted in the accumulation of both LC3B and p62, representing the blockade of the autophagic flux. In addition, inhibition of autophagy by either 3-MA or CQ blocked metformin-induced protection against H_2_O_2_-induced cell viability loss (Figures 4(b) and 4(c)). To further confirm these findings, we proceeded to the knockdown of the key autophagic proteins Beclin1 or LC3B by using shRNA. As expected, the knockdown of either gene abolished the protective effect of metformin on H_2_O_2_-induced cell viability loss, ROS production, and loss of mitochondrial membrane potential, indicating the essential role of autophagy activation in metformin-mediated protective effects (Figures 4(d)–4(h)).

### 3.5. AMPK Signaling Is Involved in the Protective Effect of Metformin against H_2_O_2_-Induced Cell Damage

**Figure 5:**
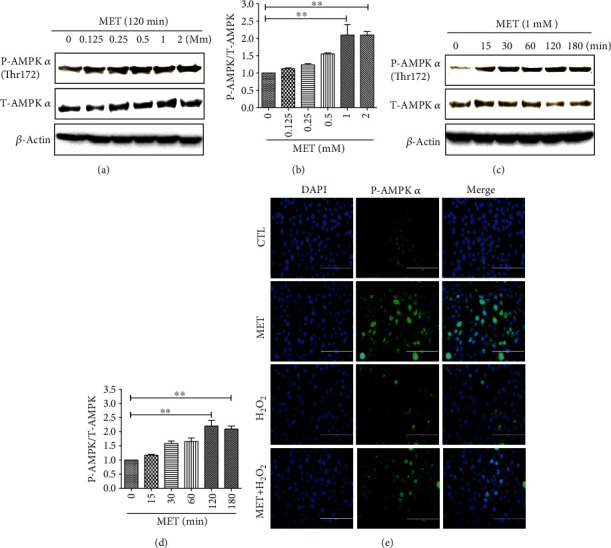
AMPK signaling is involved in the protective effect of metformin against H_2_O_2_-induced cell damage. (a) D407 cells were treated with 1 mM metformin for the periods of time indicated, and then, P-AMPK levels were assessed by Western blot. (b) Quantitative analysis of (a). (c) D407 cells were treated with different concentrations of metformin for 2 h, and then, P-AMPK levels were assessed by Western blot. (d) Quantitative analysis of (c). (e) D407 cells were pretreated with 1 mM metformin and then incubated with or without 200 *μ*M H_2_O_2_ for further 24 h. The expression of AMPK was assessed by ICC (scale bars, 200 *μ*m). Data are presented as the mean ± SD; ^∗∗^*p* < 0.01.

As metformin has been shown to activate AMPK-dependent autophagy in many tissues, we investigated whether metformin could stimulate AMPK phosphorylation in D407 cells. As shown in Figures 5(a) and 5(b), metformin treatment caused a significant upregulation of AMPK phosphorylation levels in a dose-dependent manner, starting at 1 mM. Similarly, metformin also time-dependently stimulated AMPK phosphorylation in D407 cells (Figures 5(c) and 5(d)). Additionally, we performed immunocytochemistry to detect the levels and subcellular localization of phosphorylated AMPK (P-AMPK). As presented in Figure 5(e), P-AMPK is mainly located in the cytoplasm in both basal and H_2_O_2_-stimulating conditions. Exposure to metformin promoted the increase of P-AMPK levels in both the cytoplasm and the nucleus, being more prominent in the latter, when cells were treated with or without H_2_O_2_. These findings are indicative that metformin is able to stimulate AMPK activation in D407 cells.

### 3.6. The Activation of Autophagy and the Protective Effect of Metformin Decreased after Inhibition of AMPK Signaling Pathway

**Figure 6:**
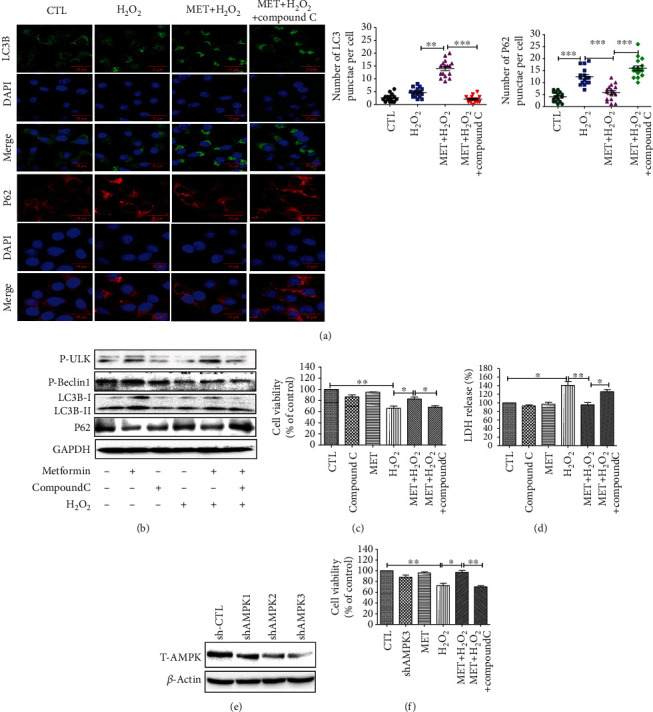
The activation of autophagy and the protective effect of metformin decreased after inhibition of the AMPK signaling pathway. D407 cells were pretreated with 2.5 *μ*M compound C for 30 min before 1 mM metformin treatment for 2 h and then incubated with or without 200 *μ*M H_2_O_2_ for further 24 h. (a) The expression of LC3B and p62 was assessed using ICC (scale bars, 50 *μ*m). (b) The expression of LC3B, p62, P-Beclin1, and P-ULK1 was assessed by Western blot. (c) Cell viability was assessed using the MTT assay. (d) Cell cytotoxicity was measured by LDH assay. (e) Cells were pretreated with shAMPK plasmid, and after 48 h, cells were harvested and shAMPK interference efficiency was detected by Western blot. (f) Cells were pretreated with shAMPK3 plasmid and then treated as described above. Data are presented as the mean ± SD; ^∗∗^*p* < 0.01, ^∗^*p* < 0.05.

In order to investigate if AMPK signaling is required for the metformin-mediated autophagy upregulation and its protective effect in D407 cells, we proceeded to the inhibition of AMPK using compound C (AMPK inhibitor). The results revealed that compound C reversed both the metformin-induced upregulation of LC3B levels and downregulation of p62 levels (Figures 6(a) and 6(b)). Moreover, AMPK inhibition blocked the effect of metformin in the phosphorylation levels of ULK-1 and Beclin1 (Figure 6(b)), suggesting that metformin-induced autophagy in D407 cells is AMPK-dependent. Further assessment of AMPK-mediated autophagy in the protective effect of metformin revealed that upon AMPK inhibition metformin failed to protect D407 cells from H_2_O_2_-induced cell viability loss (Figure 6(c)). In addition, metformin reversion of H_2_O_2_-induced LDH release was blocked by compound C treatment (Figure 6(d)). To confirm this finding, we proceeded to the knockdown of AMPK in D407 cells. The knockdown efficiency of different shRNA is shown in Figure 6(e), and shAMPK3 was used in following experiments as it was who most effectively suppressed AMPK expression. As presented in Figure 6(f), AMPK knockdown abolished metformin-mediated protection against H_2_O_2_-induced cell viability loss. Taken together, metformin conferred protection against H_2_O_2_-induced cell damage by stimulating AMPK-dependent autophagy.

### 3.7. Metformin Showed Similar Protective Effects in Primary RPE Cells

**Figure 7:**
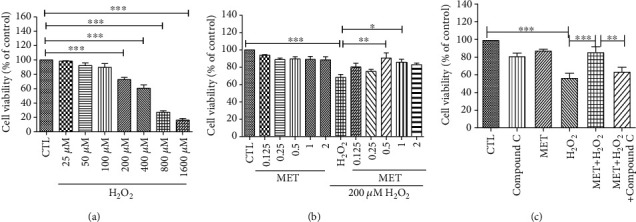
Protective effect of metformin in primary RPE cells. (a) Cells were treated with H_2_O_2_ (25-1600 *μ*M) for 24 h, and cell viability was measured using the MTT assay. (b) Cells were pretreated with metformin at the indicated concentrations and then incubated with or without 200 *μ*M H_2_O_2_ for further 24 h. (c) RPE cells were pretreated with 2.5 *μ*M compound C for 30 min before 1 mM metformin treatment for 2 h and then incubated with or without 200 *μ*M H_2_O_2_ for further 24 h. Cell viability was measured using the MTT assay. Data are presented as the mean ± SD; ^∗^*p* < 0.05, ^∗∗^*p* < 0.01, and ^∗∗∗^*p* < 0.001.

Our findings were further verified in primary RPE cells. As shown in Figure 7(a), H_2_O_2_ caused significant cytotoxicity in primary RPE cells starting at 200 *μ*M, and this concentration was chosen for the following experiments. Consistent with the observations in D407 cells, 0.125-2 *μ*M metformin treatment had no cytotoxicity effect in primary RPE cells (Figure 7(b)). Metformin pretreatment significantly reversed H_2_O_2_-induced cell viability loss and this protective effect peaked at the concentration of 0.5 mM (Figure 7(b)). In addition, compound C also blocked the protective effect of metformin against H_2_O_2_-induced cell viability loss, suggesting that metformin-mediated protection is dependent of the AMPK pathway (Figure 7(c)). Taken together, our results demonstrate that metformin has a similar protective effect in primary RPE cells.

## 4. Discussion

Although the pathogenesis of AMD is complex, RPE degeneration caused by oxidative damage has been considered a major cause of this condition. The retina is a high oxygen-consuming tissue in which redox homeostasis is particularly important [9]. Antioxidant capacity decreases with the increase of age, and the excessive ROS accumulation can lead to RPE cell damage and drive the pathogenesis of AMD [34]. Therefore, agents with antioxidant properties may confer protection against RPE degeneration induced by oxidative damage. In fact, yttrium oxide nanoparticles, which are free radical scavengers, were shown to prevent photoreceptor death in a light-damage model [35]. Additionally, dietary plant-derived antioxidants such as anthocyanins were also reported to be able to protect RPE cells from light-induced oxidative damage [36]. In the present study, we found that metformin significantly reversed H_2_O_2_-induced cell viability loss, LDH release, *Δψ*m loss, and cell apoptosis in D407 cells. As the relevant concentrations of metformin showed no cytotoxicity, we propose that metformin treatment may serve as a safe therapeutic option for preventing and treating AMD.

Autophagy is critical for protection towards oxidative damage, and diminished autophagy has been implicated in a variety of diseases including AMD. In RPE cells, inhibition of autophagy was shown to increase ROS generation and to exacerbate oxidative stress-induced mitochondria damage and cell death [7]. Thus, interventions aimed at enhancing the autophagic flux stand as a promising strategy to prevent and reverse the progression of AMD. In RPE cells, stimulation of autophagy by rapamycin was found to protect ARPE-19 cells from a lethal dose of H_2_O_2_ [7]. Despite being a potent autophagy activator, many lines of evidences have shown that rapamycin has many adverse effects, such as diabetic-like symptoms, gastrointestinal disorders, and increased risk of infection [37, 38]. Therefore, it is important to search for other alternatives with fewer side effects.

Metformin is a widely used antidiabetic drug that acts on common pathways and confers beneficial effects on a spectrum of age-related disorders. There is clinical evidence of metformin's ability to induce autophagy or prevent oxidative stress/autophagy-related diseases, including AMD. In fact, metformin was shown to be able to protect retinal photoreceptors and RPE from oxidative stress in preclinical mouse models. Moreover, its use was also reported to reduce the likelihood of developing AMD [39]. In a different study, metformin improved the neurological function and oxidative stress status of acute stroke patients with type 2 diabetes [40]. Still, the mechanisms underlying these effects remain poorly understood. In this study, we found that metformin is able to upregulate autophagy in D407 cells, as evidenced by the increased LC3B expression and decreased p62 expression. Moreover, autophagy inhibition, with either 3-MA or CQ, blocked metformin-induced changes in LC3B and p62 levels, as well as its protective effect against H_2_O_2_-mediated cell viability loss and apoptosis. The metformin protective effect was also inhibited by the knockdown of the two essential autophagic genes Beclin1 and LC3B, suggesting that metformin protects D407 cells against H_2_O_2_-induced oxidative damage by stimulating autophagy.

Metformin has been proven to be a potent AMPK activator, and its autophagy-inductive effect has been associated with AMPK activation in many tissues [41]. Consistent with these findings, our observations showed that metformin was able to stimulate AMPK phosphorylation in a time- and dose-dependent manner. The subcellular localization of AMPK is crucial for its modulatory activity on downstream targets. By immunocytochemistry staining, we found that P-AMPK was predominately located in the cytoplasm, in both basal- and H_2_O_2_-stimulating conditions. Exposure to metformin promoted the increase of P-AMPK expression in the cytoplasm and nucleus in both conditions. In addition, both pharmacological (compound C) and genetic (shRNA) inhibitions of AMPK abolished the autophagy-stimulating and protective effects of metformin, indicating that they are AMPK-dependent. Interestingly, our findings on P-AMPK nuclear localization appear to contradict those of a study reporting that although oxidative stress triggered by diethyl maleate (DEM) induced total-AMPK nuclear accumulation, the AMPK phosphorylation and the nuclear/cytosol ratio of p-AMPK were reduced [42]. One possible explanation for this discrepancy may be the use of different cell lines and reagents to induce oxidative stress. Whereas the previous study tested the effect of DEM on HeLa cells, we used H_2_O_2_ to induce oxidative stress in D407 cells. Furthermore, although metformin-dependent AMPK activation has been reported to stimulate autophagy and to have a neuroprotective role in many studies, some exceptions have also been reported. For example, in cerebral ischemia, AMPK overactivation by a large dose of metformin treatment led to prolonged astrocytic glycolysis and subsequently to exacerbated stroke injury [43–45]. In addition, metformin-mediated AMPK activation inhibited autophagy in cancer cells [46, 47]. Another study also demonstrated that AMPK activation triggered by glucose starvation was able to prevent both autophagosome formation and lysosome acidification [48]. The precise mechanisms and consequences of metformin-regulated autophagy in different cell types and conditions are an interesting question that needs further investigation.

**Figure 8:**
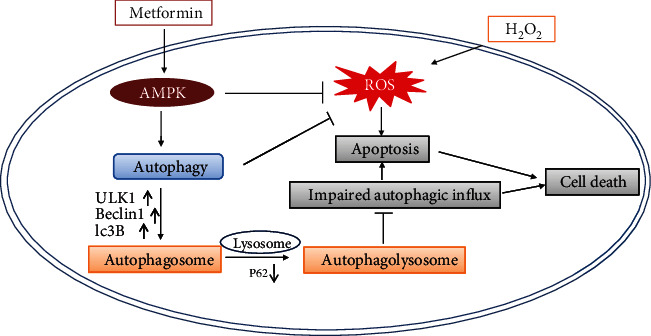
Metformin proposed the mechanism of action. Metformin activates the AMPK pathway, which further stimulates autophagy, promotes the binding of autophagosomes and lysosomes to ensure the smooth flow of autophagic flux, and prevents the cell damage caused by hydrogen peroxide.

In our study, we found that metformin could protect D407 cells from H_2_O_2_-induced oxidative damage by stimulating autophagy via the activation of AMPK pathway. In addition, metformin also conferred a similar protection in primary cultured human RPE cells. In summary, our study demonstrates that metformin is able to protect RPE cells against H_2_O_2_-induced oxidative damage, and this effect is attributed to its ability to enhance autophagy by activating the AMPK pathway (Figure 8). Our findings reveal the underlying mechanisms of the antioxidant and autophagy-modulating effects of metformin in RPE cells and support its potential use in AMD prevention and treatment.

## Figures and Tables

**Table 1:** Antibody information.

Antibody	Cat. No.	Source	Company	Dilution
LC3B (D11) XP	2639	Rabbit	CST	WB: 1 : 1000/ICC: 1 : 100
P62/SQSTM1	18420-1-AP	Rabbit	PROTEINTECH	WB: 1 : 1000/ICC: 1 : 100
P-beclin1 (ser93)	14717s	Rabbit	CST	WB: 1 : 1000
P-ulk1 (ser757)	6888s	Rabbit	CST	WB: 1 : 1000
P-AMPK*α* (Thr172)	2535s	Rabbit	CST	WB: 1 : 1000/ICC: 1 : 100
AMPK*α* (23A3)	2603S	Rabbit	CST	WB: 1 : 1000/ICC: 1 : 100
*β*-Actin (D6A8)	12620s	Rabbit	CST	WB: 1 : 1000/ICC: 1 : 100
Anti-rabbit IgG HRP	7074	Rabbit	CST	WB: 1 : 2000
Alexa Fluor® 594	8889	Rabbit	CST	ICC: 1 : 500
Alexa Fluor® 488	4412	Rabbit	CST	ICC: 1 : 500

**Table 2:** shRNA sequence information.

Gene name	GenBank ID	Target sequence	Starting position	GC (%)
shAMPK1 (PRKAA2-RNAi1)	NM_006252	GGCTTATCATCTTATCATT	1028	31.58
shAMPK2 (PRKAA2-RNAi2)	NM_006252	TGTGAAAGAAGTGTGTGAA	938	36.84
shAMPK3 (PRKAA2-RNAi3)	NM_006252	CACTGAAACGAGCAACTAT	820	42.11
shBeclin1 (BECN1)	NM_003766	gaGAGGAGCCATTTATTGAAA	358	36.84
shLC3B	NM_022818	GTGCATGTCAGTTGTGGAGAA	1653	47.62

Note: sh-CTL control insertion sequence: TTCTCCGAACGTGTCACGT; shRNA structure: hU6-MCS-Ubiquitin-EGFP-IRES-puromycin.

## Data Availability

The data used to support the findings of this study are included within the article.

## Supplementary Materials

Supplementary Materials Fig.s1: The optimal infection efficiency of the RFP-GFP-LC3 lentiviral is 80MOI. D407 cells were infected with RFP-GFP-LC3 lentivirus with different MOI (20, 40, and 80). Images were taken after 48 h by using a Nikon A1.
