# Supplementary material for: Protective Effect of Metformin against Hydrogen Peroxide-Induced Oxidative Damage in Human Retinal Pigment Epithelial (RPE) Cells by Enhancing Autophagy through Activation of AMPK Pathway
Source: Oxid Med Cell Longev. 2020 Jul 24;2020:2524174. doi: 10.1155/2020/2524174 (PMC7397438; doi:10.1155/2020/2524174)
Supplement: Supplementary Materials — Fig.s1: The optimal infection efficiency of the RFP-GFP-LC3 lentiviral is 80MOI. D407 cells were infected with RFP-GFP-LC3 lentivirus with different MOI (20, 40, and 80). Images were taken after 48 h by using a Nikon A1. [file 2524174.f1.pdf]

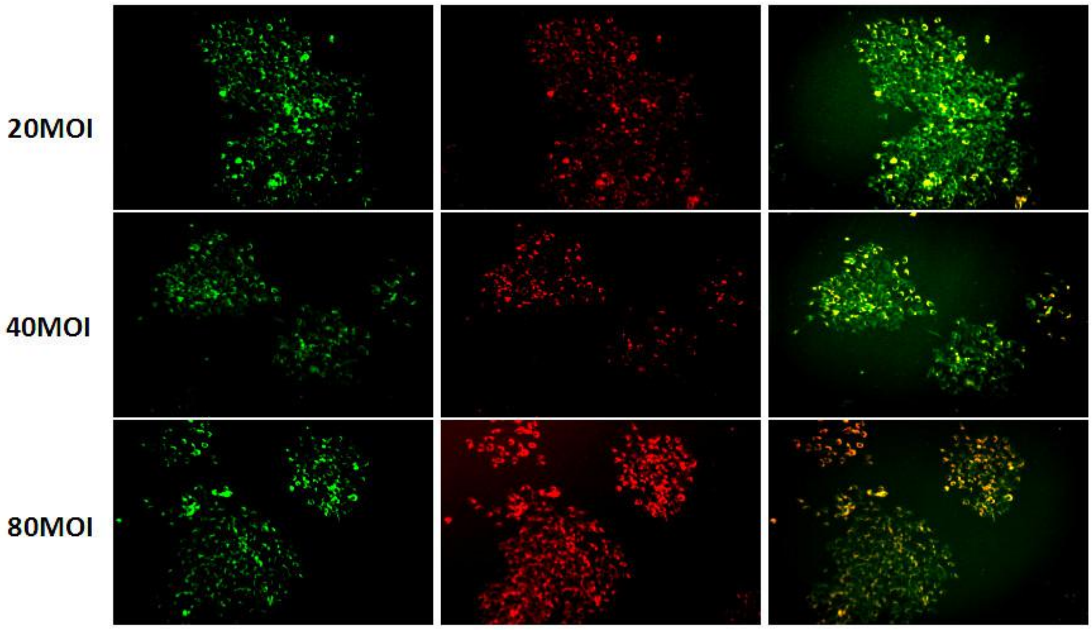

Fig.s1. The optimal infection efficiency of the RFP-GFP-LC3 lentiviral is 80MOI. D407 cells were infected with RFP-GFP-LC3 lentivirus with different MOI (20,40,80). Images were taken after 48h by using a Nikon A1 confocal microscope .
